# Supplementary material for: Real-World Use of Highly Sensitive Liquid Biopsy Monitoring in Metastatic Breast Cancer Patients Treated with Endocrine Agents after Exposure to Aromatase Inhibitors
Source: Int J Mol Sci. 2023 Jul 13;24(14):11419. doi: 10.3390/ijms241411419 (PMC10379453; doi:10.3390/ijms241411419)

SUPPLEMENTARY DOCUMENTS

Supplementary Figure S1
Supplementary Figure S2
Supplementary Figure S3
Supplementary Figure S4
Supplementary Figure S5

(Attached separately)
Supplementary Table S1. Alterations interrogated by dPCR
Supplementary Table S2. Regions interrogated by the SafeSEQ Breast Cancer Panel
Supplementary Table S3. Description and clinical significance of detected alterations in ctDNA

Supplementary Figure S1. A Higher prevalence of baseline plasma *ESR1* mutations in patients with longer exposure to aromatase inhibitors. **B** Clinical characteristics of patients with baseline *PIK3CA*, *ESR1*, and *TP53* mutations. Statistical significance highlighted in red, non-significant trends highlighted in grey.

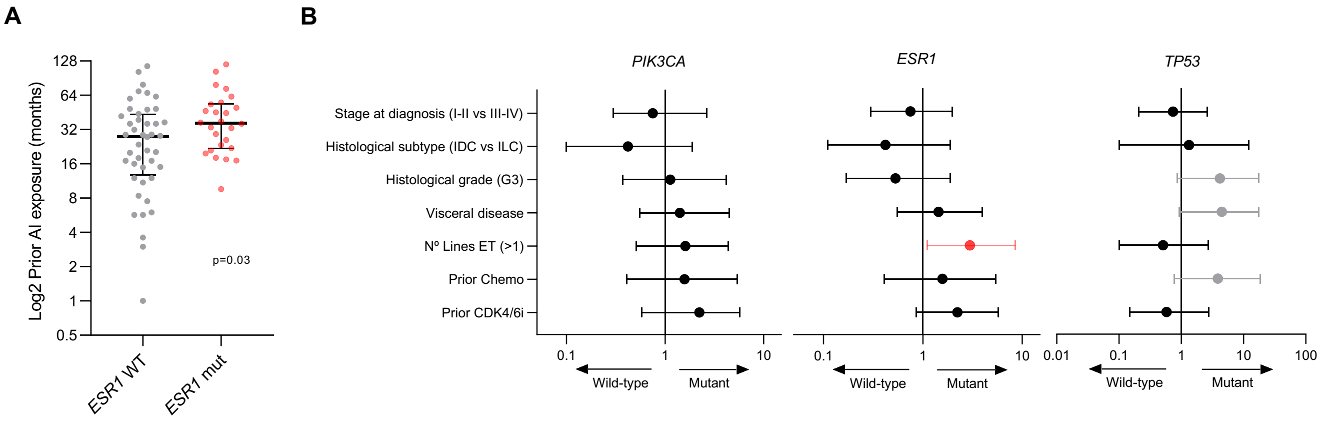

Supplementary Figure S2. A Evolution of *PIK3CA* (green) and *ESR1* (red) mutation prevalence across primary, metastasis, and baseline and progression plasma samples. VAF is split by ≥0.1% and <0.1%. **B** Landscape of gene mutations and their VAF in patients with available SafeSEQ data from baseline plasma samples (top panel). Only patients with a pathogenic mutation in baseline plasma are shown. *PIK3CA* and *ESR1* mutations identified by dPCR in tissue samples (bottom panel). VAFs lower than 0.1% are shadowed. **C** Distribution of DNA content according to VAF. **D, E** Venn diagrams illustrating the concordance in *PIK3CA* (up) and *ESR1* (down) mutation detection between tissue and plasma, considering all mutations (left) and those with VAF≥1% (right)

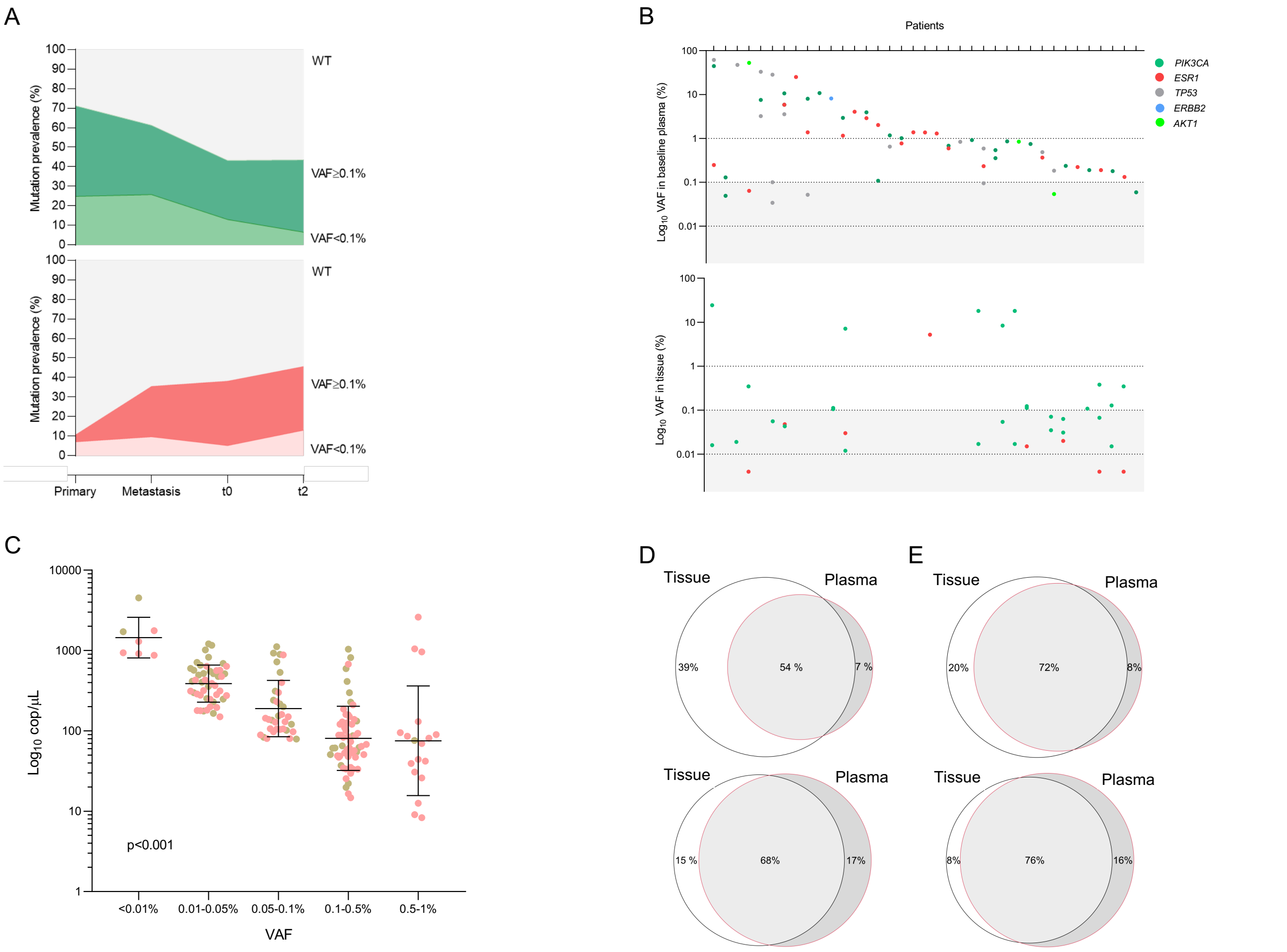

Supplementary Figure S3. A Distribution of cfDNA levels early on treatment according to best response by RECIST 1.1. **B** Distribution of cfDNA levels early on treatment according to progression-free survival, stratified by early progressors (<4.5 months) and late progressors (>9 months)

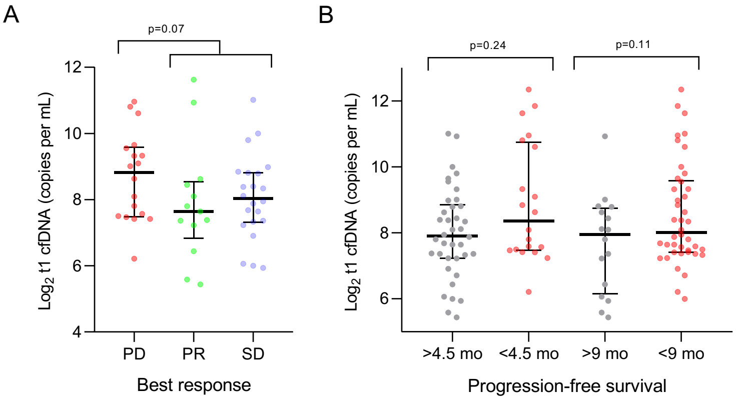

Supplementary Figure S4. A-D Impact of baseline plasma *PIK3CA* mutational status in the progression-free survival of patient receiving SERD monotherapy (**A**), aromatase inhibitor monotherapy (**B**), combined endocrine therapy and CDK4/6 inhibitors (**C**), and combined exemestane and everolimus (**D**). **E-H** Impact of baseline plasma *ESR1* mutational status in the above-mentioned subgroups. **I** Influence of the VAF in the prognostic role of *PIK3CA* mutational status. **J, K** Progression-free survival of patients with baseline plasma *PIK3CA* and *ESR1* mutations according to the type of endocrine therapy. **L** Progression-free survival among patients with baseline individual or concurrent mutations, or none.

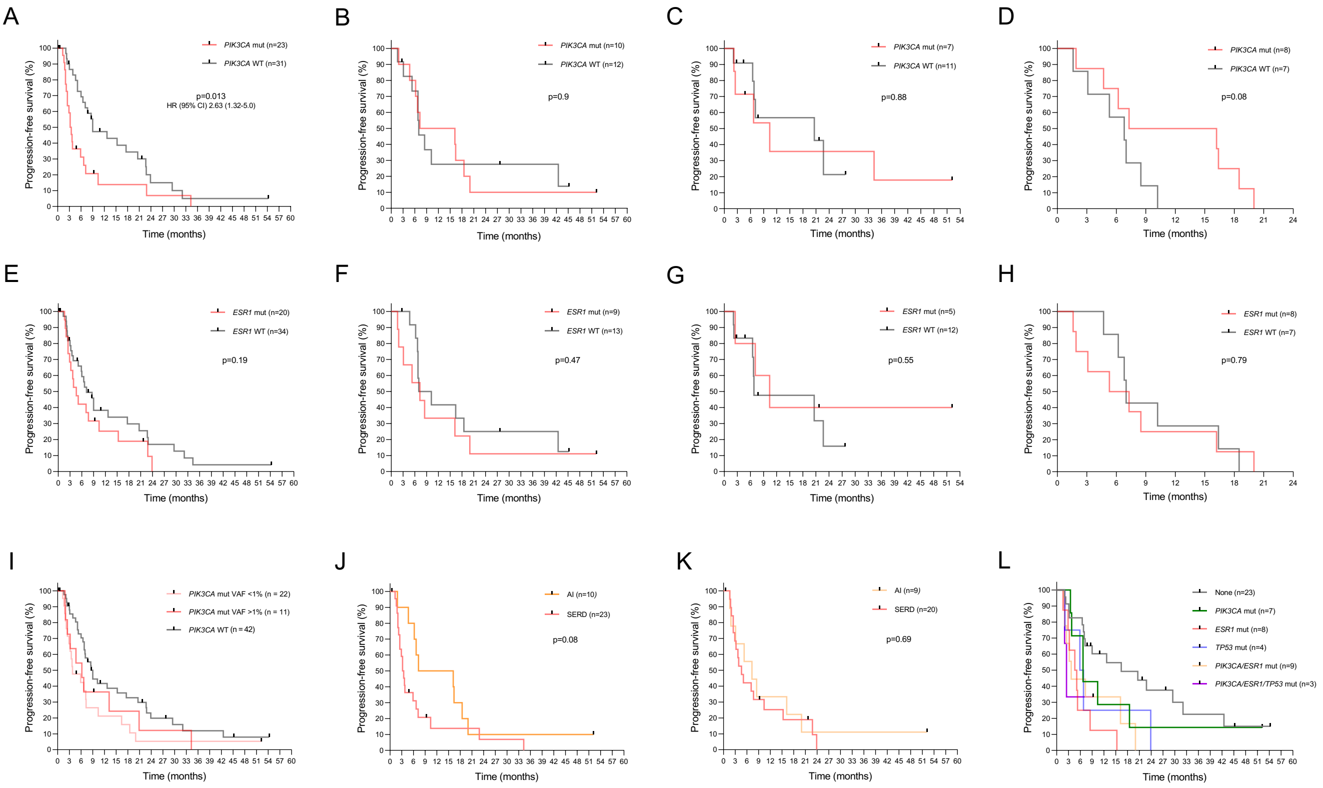

Supplementary Figure S5. A,B Impact of early mutant *PIK3CA* dynamics in patients receiving SERD- **(A)** or aromatase inhibitor-containing regimens **(B). C,D** Impact of early mutant *ESR1* dynamics in patients receiving SERD- **(C)** or aromatase inhibitor-containing regimens **(D).**

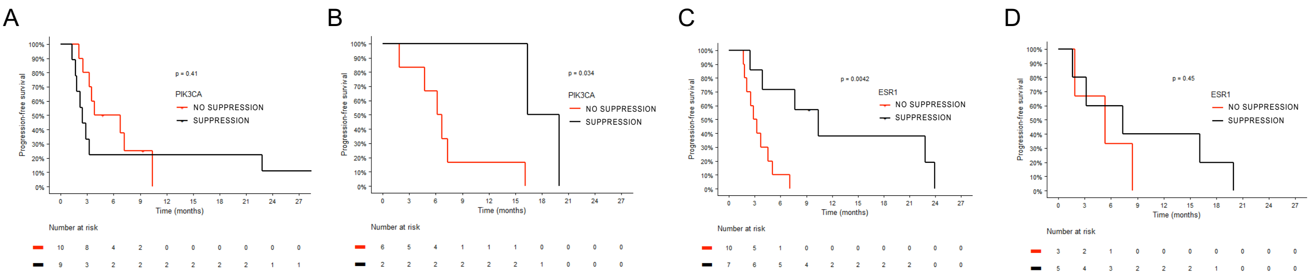

Supplement: Supplementary file 1 [file ijms-24-11419-s001.zip › Supplementary Figures.pdf]
