# Supplementary material for: Real-World Use of Highly Sensitive Liquid Biopsy Monitoring in Metastatic Breast Cancer Patients Treated with Endocrine Agents after Exposure to Aromatase Inhibitors
Source: Int J Mol Sci. 2023 Jul 13;24(14):11419. doi: 10.3390/ijms241411419 (PMC10379453; doi:10.3390/ijms241411419)
Supplement: Supplementary file 1 [file ijms-24-11419-s001.zip › Supplementary Table S1.pdf]

Supplementary Table S1. Interrogated alterations by dPCR

Gene	Nucleotide_change	Amino_acid_change	COSMIC_ID	Assay_ID	Assay_name	wild type allele (VIC label)	Mutant allele (FAM label)	Foward Primer	Reverse Primer	MGB Probes (wt/mutant)
PIK3CA	c.1624G>A	E542K	COSV55873227	Hs000000085_rm	PIK3CA_760	G	A	NA	NA	NA
PIK3CA	c.1633G>A	E545K	COSV55873239	Hs000000086_rm	PIK3CA_763	G	A	NA	NA	NA
PIK3CA	c.3140A>G	H1047R	COSV55873195	Hs000000088_rm	PIK3CA_775	A	G	NA	NA	NA
ESR1	c.1610A>C	Y537S	COSV52783938	AH1SEW5_custom	NA	A	C	5´-CTGTACAGCATGAAGTGCAAGAAC-3´	5´-GGCTAGTGGGCGCATGTAG-3´	5’-TGCCCCTCT***A*** TGACCTGC-3’ / 5’-TGCCCCTCT***C*** TGACCTGC-3’
ESR1	c.1613A>G	D538G	COSV52781024	AH519FT_custom	NA	A	G	5´-CTGTACAGCATGAAGTGCAAGAAC-3´	5´-GGCTAGTGGGCGCATGTAG-3´	5’-CCCTCTATG***A*** CCTGCTGC-3’ / 5’-CCCTCTATG***G*** CCTGCTGC-3
