# Supplementary material for: Real-World Use of Highly Sensitive Liquid Biopsy Monitoring in Metastatic Breast Cancer Patients Treated with Endocrine Agents after Exposure to Aromatase Inhibitors
Source: Int J Mol Sci. 2023 Jul 13;24(14):11419. doi: 10.3390/ijms241411419 (PMC10379453; doi:10.3390/ijms241411419)
Supplement: Supplementary file 1 [file ijms-24-11419-s001.zip › Supplementary Table S2.pdf]

Supplementary Table S2. Regions interrogated by the SafeSEQ Breast Cancer Panel. GRCh38.p13

Gene	Transcript	Amino Acid Start	Amino Acid End	Primary clinical relevance
**ESR1**	ENST00000440973	370	381	Acquired after hormone therapy, putative predictive marker for novel ESR1-directed therapies
		460	473	
		529	538	
**PIK3CA**	ENST00000263967	86	92	Frequently mutated for ER+/HER2- BC, investigational therapies targeting the PIK/AKT1 pathway
		111	117	
		119	122	
		345	352	
		363	371	
		418	421	
		450	462	
		538	553	
		714	728	
		1040	1056	
**AKT1**	ENST00000349310	17	23	Investigational therapies targeting the PI3K/AKT1 pathway
**ERBB2**	ENST00000269571	303	315	Emerging resistance mechanism for ESR1-directed therapies
		754	769	
		770	786	
**KRAS**	ENST00000311936	4	14	Emerging potential resistance mutation for hormone therapy
**TP53**	ENST00000269305	49	77	Frequently mutated for ER+/HER2- BC, "truncal" mutation which can be used to determine presence/absence of detectable ctDNA in the blood
		99	125	
		126	141	
		151	179	
		192	219	
		233	260	
		262	285	
		297	306	
		308	331	
		332	360	
