# Supplementary material for: Real-World Use of Highly Sensitive Liquid Biopsy Monitoring in Metastatic Breast Cancer Patients Treated with Endocrine Agents after Exposure to Aromatase Inhibitors
Source: Int J Mol Sci. 2023 Jul 13;24(14):11419. doi: 10.3390/ijms241411419 (PMC10379453; doi:10.3390/ijms241411419)
Supplement: Supplementary file 1 [file ijms-24-11419-s001.zip › Supplementary Table S3.pdf]

Supplementary Table S3. Description and clinical significance of detected alterations in ctDNA. *In silico* predictors (Sift, PolyPhen and FATHMM-MKL) for missense variants were used in those cases without ClinVar information or with inconclusive result GRCh38

Gene Name	Transcript	CDS Change	AA Change	Consequence	Analysis	COSMIC_ID	ClinVar_ID	ClinVar_Significance	Sift	PolyPhen	FATHMM-MKL
PIK3CA	ENST00000263967	c.1035T>A	p.N345K	Missense variant	SafeSeq	COSV55873276	376050	Pathogenic	-	-	-
PIK3CA	ENST00000263967	c.1258T>C	p.C420R	Missense variant	SafeSeq	COSV55874020	31945	Pathogenic	-	-	-
PIK3CA	ENST00000263967	c.1346_1366del	p.P449_L455del	Inframe deletion	SafeSeq	COSV55874439	NA	-	-	-	-
PIK3CA	ENST00000263967	c.1624G>C	p.E542Q	Missense variant	SafeSeq	COSV55894248	376244	Pathogenic;Likely_pathogenic	-	-	-
PIK3CA	ENST00000263967	c.1624G>A	p.E542K	Missense variant	dPCR + SafeSeq	COSV55873227	31944	Pathogenic;Likely_pathogenic	-	-	-
PIK3CA	ENST00000263967	c.1633G>A	p.E545K	Missense variant	dPCR + SafeSeq	COSV55873239	13655	Pathogenic;Likely_pathogenic	-	-	-
PIK3CA	ENST00000263967	c.1636C>A	p.Q546K	Missense variant	SafeSeq	COSV55873527	13657	Pathogenic;Likely_pathogenic	-	-	-
PIK3CA	ENST00000263967	c.2176G>A	p.E726K	Missense variant	SafeSeq	COSV55875460	376476	Pathogenic	-	-	-
PIK3CA	ENST00000263967	c.3129G>T	p.M1043I	Missense variant	SafeSeq	COSV55878974	217292	Pathogenic	-	-	-
PIK3CA	ENST00000263967	c.3140A>T	p.H1047L	Missense variant	SafeSeq	COSV55873401	13653	Pathogenic	-	-	-
PIK3CA	ENST00000263967	c.3140A>G	p.H1047R	Missense variant	dPCR + SafeSeq	COSV55873195	13652	Pathogenic	-	-	-
ESR1	ENST00000440973	c.1138G>C	p.E380Q	Missense variant	SafeSeq	COSV52782264	376206	Likely_pathogenic	-	-	-
ESR1	ENST00000440973	c.1610A>C	p.Y537S	Missense variant	dPCR + SafeSeq	COSV52783938	NA	-	deleterious	probably damaging	pathogenic
ESR1	ENST00000440973	c.1609T>A	p.Y537N	Missense variant	SafeSeq	COSV52784978	NA	-	deleterious	probably damaging	pathogenic
ESR1	ENST00000440973	c.1610A>G	p.Y537C	Missense variant	SafeSeq	COSV52782924	NA	-	deleterious	probably damaging	pathogenic
ESR1	ENST00000440973	c.1613A>G	p.D538G	Missense variant	dPCR + SafeSeq	COSV52781024	NA	-	deleterious	probably damaging	pathogenic
TP53	ENST00000269305	c.182insT	p.62E>*_fs	Frameshift variant	SafeSeq	NA	NA	-	-	-	-
TP53	ENST00000269305	c.215C>G	p.P72R	Missense variant	SafeSeq	COSV52666208	12351	benign	-	-	-
TP53	ENST00000269305	c.329G>C	p.R110P	Missense variant	SafeSeq	COSV52668419	233627	Pathogenic	-	-	-
TP53	ENST00000269305	c.331_333delinsTC	p.L111Sfs*12	Frameshift variant	SafeSeq	COSV53606624	NA	-	-	-	-
TP53	ENST00000269305	c.337T>A	p.F113I	Missense variant	SafeSeq	COSV53351693	NA	-	deleterious	probably damaging	pathogenic
TP53	ENST00000269305	c.354A>T	p.T118=	Synonymous variant	SafeSeq	COSV53220114	NA	-	-	-	-
TP53	ENST00000269305	c.394A>G	p.K132E	Missense variant	SafeSeq	COSV52689323	376626	Conflicting_interpretations_of_pathogenicity	deleterious	probably damaging	pathogenic
TP53	ENST00000269305	c.404G>T	p.C135F	Missense variant	SafeSeq	COSV52680475	376559	Conflicting_interpretations_of_pathogenicity	deleterious	probably damaging	pathogenic
TP53	ENST00000269305	c.466_469delinsTCC	p.R156Sfs*14	Frameshift variant	SafeSeq	COSV52990383	NA	-	-	-	-
TP53	ENST00000269305	c.475G>C	p.A159P	Missense variant	SafeSeq	COSV52661847	182929	Uncertain_significance	deleterious	probably damaging	pathogenic
TP53	ENST00000269305	c.490A>G	p.K164E	Missense variant	SafeSeq	COSV52728094	246416	Uncertain_significance	deleterious	probably damaging	pathogenic
TP53	ENST00000269305	c.532C>G	p.H178D	Missense variant	SafeSeq	COSV52693338	482223	Likely_pathogenic			
TP53	ENST00000269305	c.578A>T	p.H193L	Missense variant	SafeSeq	COSV52663304	185822	Conflicting_interpretations_of_pathogenicity	deleterious	probably damaging	pathogenic
TP53	ENST00000269305	c.578A>G	p.H193R	Missense variant	SafeSeq	COSV52662414	184979	Pathogenic;Likely_pathogenic			
TP53	ENST00000269305	c.583A>T	p.I195F	Missense variant	SafeSeq	COSV52677568	376617	Conflicting_interpretations_of_pathogenicity	deleterious	probably damaging	pathogenic
TP53	ENST00000269305	c.639A>G	p.R213=	Synonymous variant	SafeSeq	COSV52679610	NA	-	-	-	-
TP53	ENST00000269305	c.658T>C	p.Y220H	Missense variant	SafeSeq	COSV52760651	376687	Conflicting_interpretations_of_pathogenicity	deleterious	probably damaging	pathogenic
TP53	ENST00000269305	c.707A>G	p.Y236C	Missense variant	SafeSeq	COSV52662150	376693	Conflicting_interpretations_of_pathogenicity	deleterious	probably damaging	pathogenic
TP53	ENST00000269305	c.711G>A	p.M237I	Missense variant	SafeSeq	COSV52661887	142714	Pathogenic	-	-	-
TP53	ENST00000269305	c.716A>G	p.N239S	Missense variant	SafeSeq	COSV52661127	376637	likely_pathogenic	-	-	-
TP53	ENST00000269305	c.730G>A	p.G244S	Missense variant	SafeSeq	COSV52676997	376600	Pathogenic	-	-	-
TP53	ENST00000269305	c.743G>A	p.R248Q	Missense variant	SafeSeq	COSV52661580	12356	Pathogenic	-	-	-
TP53	ENST00000269305	c.757A>C	p.T253P	Missense variant	SafeSeq	COSV52873550	NA	-	deleterious	probably damaging	pathogenic
TP53	ENST00000269305	c.764T>A	p.I255N	Missense variant	SafeSeq	COSV52714133	232289	Uncertain_significance	deleterious	probably damaging	pathogenic
TP53	ENST00000269305	c.799C>T	p.R267W	Missense variant	SafeSeq	COSV52678166	141764	Pathogenic;Likely_pathogenic	-	-	-
TP53	ENST00000269305	c.817C>T	p.R273C	Missense variant	SafeSeq	COSV52662066	43594	Pathogenic;Likely_pathogenic	-	-	-
TP53	ENST00000269305	c.818G>T	p.R273L	Missense variant	SafeSeq	COSV52664805	376655	Pathogenic	-	-	-
TP53	ENST00000269305	c.839G>C	p.R280T	Missense variant	SafeSeq	COSV52677783	12368	Uncertain_significance	deleterious	probably damaging	pathogenic
TP53	ENST00000269305	c.991C>T	p.Q331*	Nonsense variant	SafeSeq	COSV52664188	922893	Pathogenic	-	-	-
TP53	ENST00000269305	c.1018A>T	p.M340L	Missense variant	SafeSeq	NA	NA	-	tolerated	benign	pathogenic
AKT1	ENST00000349310	c.47-30G>A	p.?	Missense variant	SafeSeq	NA	NA	-			
AKT1	ENST00000349310	c.49G>A	p.E17K	Missense variant	SafeSeq	COSV62571334	15017	Pathogenic	-	-	-
ERBB2	ENST00000269571	c.937C>G	p.L313V	Missense variant	SafeSeq	COSV54067264	NA	-	tolerated	benign	pathogenic
KRAS	ENST00000311936	c.34G>T	p.G12C	Missense variant	SafeSeq	COSV55497469	12578	pathogenic	-	-	-
KRAS	ENST00000311936	c.35G>T	p.G12V	Missense variant	SafeSeq	COSV55497419	12583	pathogenic;likely_pathogenic	-	-	-
